# The Influence of Metabolism on Drug Response in Cancer

**DOI:** 10.3389/fonc.2018.00500

**Published:** 2018-11-02

**Authors:** Esther A. Zaal, Celia R. Berkers

**Affiliations:** ^1^Biomolecular Mass Spectrometry and Proteomics, Bijvoet Center for Biomolecular Research, Utrecht University, Utrecht, Netherlands; ^2^Department of Biochemistry and Cell Biology, Faculty of Veterinary Medicine, Utrecht University, Utrecht, Netherlands

**Keywords:** cancer metabolism, drug resistance, bortezomib, cisplatin, BRAF inhibitors, multiple myeloma, melanoma, breast cancer

## Abstract

Resistance to therapeutic agents, either intrinsic or acquired, is currently a major problem in the treatment of cancers and occurs in virtually every type of anti-cancer therapy. Therefore, understanding how resistance can be prevented, targeted and predicted becomes increasingly important to improve cancer therapy. In the last decade, it has become apparent that alterations in cellular metabolism are a hallmark of cancer cells and that a rewired metabolism is essential for rapid tumor growth and proliferation. Recently, metabolic alterations have been shown to play a role in the sensitivity of cancer cells to widely-used first-line chemotherapeutics. This suggests that metabolic pathways are important mediators of resistance toward anticancer agents. In this review, we highlight the metabolic alterations associated with resistance toward different anticancer agents and discuss how metabolism may be exploited to overcome drug resistance to classical chemotherapy.

## Introduction

Changes in metabolism are one of the emerging hallmarks of cancer cells (1). Although many signaling pathways that are affected by genetic mutations in cancer influence metabolism (2), metabolic alterations are more than just an epiphenomenon (3). Alterations in cellular metabolism sustain rapid production of adenosine triphosphate (ATP) and increased biosynthesis of macromolecules, including nucleotides, lipids and amino acids, and also help maintain cellular redox state (2, 4). As such, a rewired metabolism is essential to meet the needs of tumors for rapid cell growth and proliferation.

Both intrinsic and extrinsic mechanisms contribute to the characteristic metabolic alterations in cancer cells. Many different oncogenic as well as tumor suppressor signaling pathways influence metabolism, such as hypoxia-inducible factor 1 (HIF1), p53 and MYC. In addition, cancer metabolism is influenced by the tumor microenvironment, for example the interaction with surrounding cells and the variation in availability of nutrients and oxygen, as extensively reviewed elsewhere (2, 5–12). These mechanisms affect pathways involved in central carbon metabolism, such as glycolysis and the tricarboxylic acid (TCA) cycle, amongst others. As a result, cancer cells have an increased consumption of glucose and glutamine to satisfy their altered metabolic needs. The fact that cancer cells can become addicted to specific metabolic pathways has led to the recent development of novel drugs that target these metabolic vulnerabilities (13, 14).

Resistance to therapeutic agents, either intrinsic or acquired, is currently a major problem in the treatment of cancers and occurs in virtually every type of anti-cancer therapy (15, 16). Although increased knowledge about the molecular mechanisms of cancer has led to the development of novel targeted therapeutic compounds that increase progression-free survival, this does not always translate in overall survival benefits due to development of resistance (17). Acquired drug resistance can result from the acquisition of mutations causing decreased drug binding, increased activity of the drug target or the upregulation of multi-drug resistance transporters. Acquired resistance can also be the result of various adaptive responses that occur downstream of the drug target and that help cancer cells withstand the effects of the drug [reviewed in (18)]. Examples of such mechanisms are the upregulation of cellular pro-survival pathways, including the activation of DNA repair mechanisms (19), the upregulation of anti-apoptotic proteins (20, 21) or autophagy (22). Another mechanism of resistance, that is frequently observed with kinase inhibitor therapy, is the so-called “oncogenic bypass,” in which the target pathway is activated through an alternative kinase, even when the primary kinase remains inhibited (23–26). Although adaptive resistance can be targeted to improve drug efficacy, heterogeneity and adaptability of cancer cells often leads to new forms of adaptive resistance (27). Therefore, understanding how resistance can be prevented, targeted, and predicted becomes increasingly important to improve cancer therapy.

Recent studies show that the response to widely-used first-line chemotherapy is substantially influenced by the metabolic state of the cells and that cancer cells rewire their metabolism in response to chemotherapeutic drugs. We postulate that metabolic rewiring is a novel and important mechanism of adaptive resistance. Here, we will introduce the main features of cancer metabolism in relation to drug resistance and review specific metabolic programs and adaptations that exist in drug-resistant tumors. We will discuss how these adaptations depend both on the drug and the origin of the tumor and how they contribute to drug resistance, focussing on widely-used chemotherapeutics, including proteasome inhibitors (multiple myeloma), EGFR inhibitors (breast cancer), cisplatin (lung cancer/ovarian cancer) and BRAF inhibitors (melanoma). Finally, we will illustrate how targeting metabolism could overcome drug resistance to standard chemotherapy.

## Cancer metabolism

### Changes in metabolism are essential to sustain cancer cell growth and proliferation

**Figure 1:**
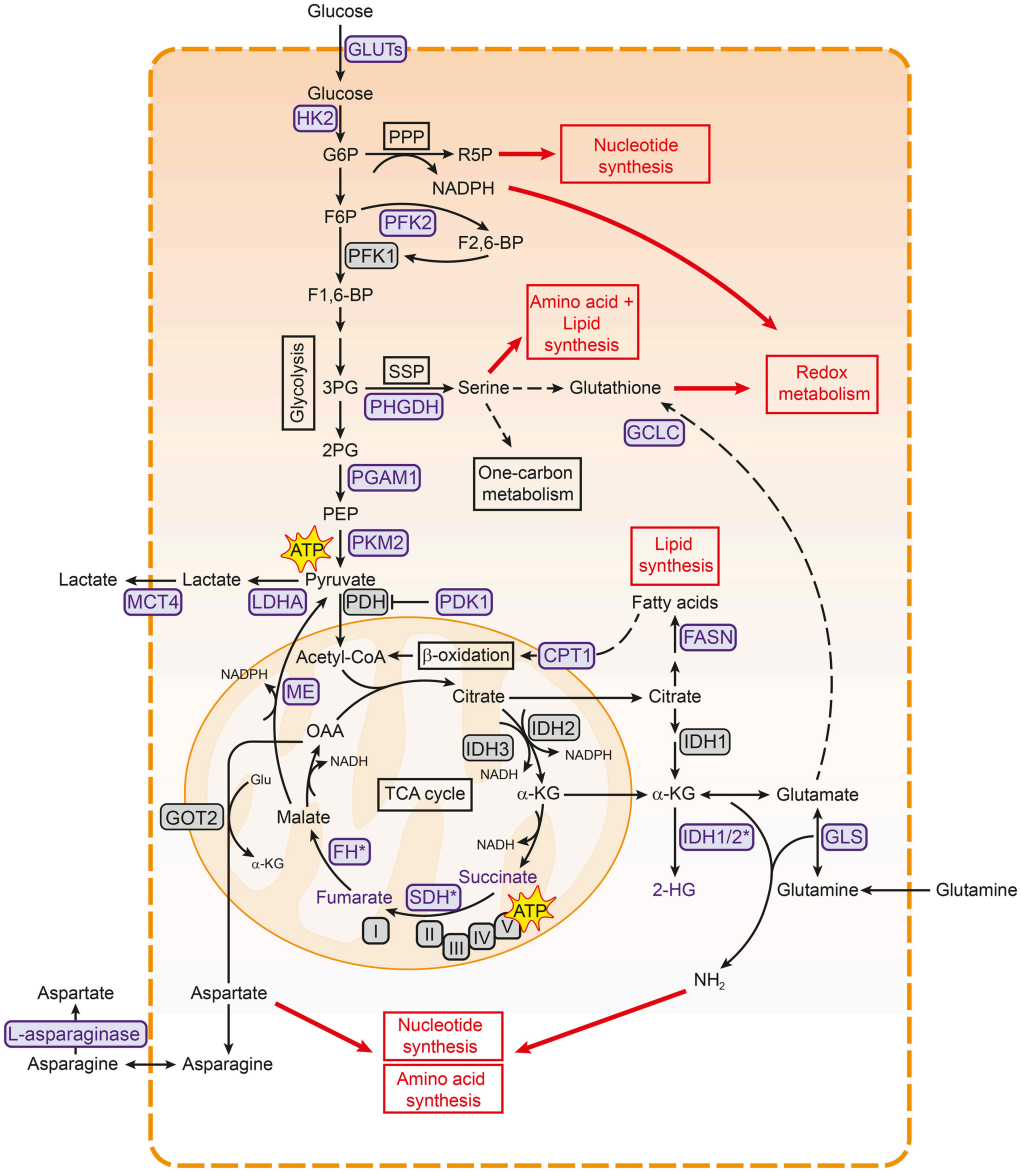
Metabolic pathways associated with cancer. Pathways involved in central carbon metabolism are presented. Metabolic enzymes that are often upregulated in cancer and serve as potential therapeutic targets are shown in purple. These metabolic pathways are involved in the synthesis of building blocks for macromolecules and redox homeostasis, needed for cell proliferation (shown in red boxes). 2PG, 2-phosphoglycerate; 3PG, 3-phoshoglycerate; ATP, adenosine triphosphate; CPT1, carnitine palmitoyltransferase I; F1,6-BP, fructose-1,6-bisphosphate; F2,6-BP, fructose-2,6-bisphosphate; F6P, fructose- 6-phosphate; FASN, fatty acid synthase; FH, fumarase; G6P, glucose-6-phosphate; GCLC, glutamate-cysteine ligase; GLS, glutaminase; Glu, glutamate; GLUT, glucose transporter type; HK2, hexokinase 2; I, complex I; IDH, isocitrate dehydrogenase; II, complex II; III, complex III; IV, complex IV; LDHA, lactate dehydrogenase A; MCT4, monocarboxylate transporter 4; ME, malic enzyme; OAA, oxaloacetate; PDH, pyruvate dehydrogenase complex; PDK1, pyruvate dehydrogenase kinase 1; PEP, phosphoenol pyruvate; PFK1, phosphofructokinase 1; PFK2, phosphofructokinase 2; PGAM1, phosphoglycerate mutase 1; PHGDH, 3-phosphoglycerate dehydrogenase; PKM2, pyruvate kinase M2; PPP, pentose phosphate pathway; R5P, ribose 5-phosphate; SDH, succinate dehydrogenase; SSP, serine synthesis pathway; TCA, tricarboxylic acid cycle; V, complex V.

Glycolysis is the main pathway that is responsible for the breakdown of glucose, and converts glucose to pyruvate in several steps (Figure 1). Glycolysis results in the production of a limited amount of energy in the form of ATP and reducing equivalents in the form of NADH. Pyruvate can subsequently be fed into the mitochondrial TCA cycle, where it is condensed with oxaloacetate to produce citrate. A series of subsequent reactions yields reducing equivalents in the form of NADH and FADH_2_, which can be oxidized in the electron transport chain (ETC) complexes to ultimately produce ATP in a process called oxidative phosphorylation (OXPHOS) (28, 29) (Figure 1). Although ATP production via OXPHOS is more efficient, the majority of cancer cells generate most of their ATP through glycolysis, even in the presence of oxygen (30). This phenomenon is known as aerobic glycolysis or “the Warburg effect” and is characterized by an increased glycolytic rate, whereby pyruvate is converted to lactate and secreted by the cell instead of being funneled into the TCA cycle.

Cancer cells sustain their high glycolytic rates in several ways. For example, glycolytic cancers often meet the high demand for extracellular glucose by overexpression of glucose transporters (GLUTs) (31, 32). They also show higher levels of monocarboxylate transporter 4 (MCT4), which is responsible for lactate export and thereby helps both in maintaining intracellular pH and in continuing glycolysis (33, 34). In addition, the secretion of lactate could aid in creating an acidic extracellular tumor environment that favors tumor growth by promoting migration and invasion (35, 36). Interestingly, cancer cells seem to rely more on specific isoforms of glycolytic enzymes, making these promising targets to specifically inhibit glycolysis in cancer cells (Figure 1) (14). For example, the M2 isoform of pyruvate kinase (PKM2) is preferentially expressed over other isoforms in most cancer cells (37). PKM2 catalyzes the final step in glycolysis, and cancer cells are thought to regulate its activity to either increase glycolytic rates or divert glycolytic intermediates to biosynthetic pathways (38), as detailed below. Cancers can also be more dependent on isoforms of hexokinase (HK2) (39, 40) and lactate dehydrogenase (LDHA) (41), or overexpress an isoform of phosphoglycerate mutase (PGAM1) (42, 43) (Figure 1). Finally, several metabolic enzymes that regulate glycolysis are highly expressed in cancer, including pyruvate dehydrogenase kinase 1 (PDK1) (44) and phosphofructokinase 2 (PFK2) (45, 46), allowing cancer cells to easily adapt glycolytic flux to meet their needs.

### Diverting glycolytic resources toward the production of building blocks

Why cancer cells prefer the less efficient glycolysis over OXPHOS for ATP production is not fully understood. Initially, Warburg hypothesized that cancer cells increase glycolytic activity because of impaired mitochondrial function (30). Indeed, several cancers are associated with mutations in TCA cycle enzymes, supporting this hypothesis (47, 48) (Figure 1). However, cancer cells also prefer glycolysis when mitochondrial function is intact (49, 50), suggesting that glycolysis confers other advantages to cancer cells. As several glycolytic intermediates can branch off into key biosynthetic pathways to generate nucleotides, amino acids and fatty acids, one of the main functions of the increased glycolytic rates is likely to meet the increased biosynthetic needs of cancer cells (4).

One of the pathways that branches of glycolysis is the pentose phosphate pathway (PPP), which both sustains the biosynthesis of macromolecules and maintains redox homeostasis (Figure 1) (51–53). The PPP produces ribose-5-phosphate for nucleotide synthesis, and regenerates NADPH to provide reducing power for glutathione and thioredoxin, both of which can capture the reactive oxygen species (ROS) that are produced during rapid cell proliferation. Glucose-6-phosphate dehydrogenase (G6PD), which catalyzes the first step of the PPP, is upregulated in numerous cancer cells, underlining the importance of the PPP in cancer metabolism (53, 54). In addition, the glycolytic intermediate 3-phosphoglycerate is used for the synthesis of the non-essential amino acid serine and downstream metabolites through the serine synthesis pathway (SSP; Figure 1). The SSP has emerged as a key pathway in cancer metabolism. Serine is needed to synthesize reduced glutathione and phospholipids and also plays a major role in the one-carbon cycle, which sustains both the biosynthesis of nucleotides and NADPH regeneration (55, 56). In line with this role, the SSP is often highly active in cancer cells and 3-phoshoglycerate dehydrogenase (PHGDH), the first and rate-limiting enzyme in this pathway, is frequently upregulated in different cancers (57, 58). The influx of glycolytic intermediates into the SSP and PPP can also be regulated by the glycolytic enzymes PKM2 (59–62) and PGAM1 (63). Lower activity of these enzymes results in accumulation of upstream metabolites, which then enter the SSP and PPP. Cancer cells thus employ various strategies to tune the diversion of glycolytic metabolites into biosynthetic pathways, underscoring the importance of glycolytic regulators in cancer metabolism (4).

### Rewiring of glutamine metabolism in the mitochondria

As intermediates of the TCA cycle are also building blocks for the biosynthesis of lipids and nucleotides (Figure 1), the TCA cycle is as important as glycolysis for cancer cell anabolism. Citrate can be used for fatty acid synthesis via fatty acid synthase (FASN), which has shown to be important in cancer cells [reviewed in (64)]. Aspartate, which is synthesized from oxaloacetate and glutamate, is important for nucleotide synthesis, making the TCA cycle important for DNA synthesis (65). In addition, malate can exit the TCA cycle via malic enzyme (ME), resulting in the production of NADPH (Figure 1) (66).

Because many TCA intermediates are shuttled into biosynthetic pathways, a new supply of carbons is needed to maintain TCA cycle activity, a process called anaplerosis. One of the most important anaplerotic pathways in cancer is glutaminolysis, in which glutamine is used to replenish the TCA cycle. Indeed, glutamine is the second most consumed metabolite in proliferating cells in cell culture (67, 68). It has been shown that glutamine is needed for protein-, fatty acid-, and nucleotide synthesis, but is also important for redox homeostasis and protein *O*-GlcNAcylation (66, 69, 70). As a result, many tumor cells are more dependent on glutamine as compared to healthy cells (71, 72).

After glutamine enters the cell, it is converted to glutamate by glutaminase (GLS). Glutamate in turn can be further converted to α-ketoglutarate that can subsequently enter the TCA cycle (Figure 1). Via glutamate, glutamine is used for the production of the amino acids aspartate and proline. Both these amino acids can be limiting for proliferation in cancer cells (65, 73, 74). In addition, glutathione cysteine ligase (GCLC), which converts glutamate to GSH, is highly expressed in several cancers (75, 76). These examples underscore the importance of glutamine and downstream pathways in cancer growth.

However, a range of other metabolites has also been described to fuel the TCA cycle in cancer. Fatty acids are not only important components of membranes, but are also energy-rich compounds that can be degraded to provide ATP via β-oxidation (77). Carnitine palmitoyl transferase 1 (CPT1) conjugates fatty acids with carnitine to translocate them to mitochondria, where β-oxidation takes place (Figure 1). CPT1C, an atypical isoform of CPT1, is highly expressed in cancers and promotes β-oxidation and ATP production (78). In addition, lactate (79), acetate (80), and branched chain amino acids (BCAA) (81) can provide the TCA cycle with carbons, illustrating the complexity of cancer metabolism.

Interestingly, TCA cycle enzymes are emerging as mediators of malignant transformation in cancer. Fumarate hydratase (FH) and succinate dehydrogenase (SDH) are tumor suppressors (Figure 1). Loss-of-function mutations in these genes are associated with tumorigenesis [reviewed in (82)] and result in the accumulation of succinate and fumarate, respectively, both of which function as oncometabolites (83, 84). Mutations in isocitrate dehydrogenase 1 (IDH1) and IDH2 are present in many cancers (85, 86) and result in the production of the oncometabolite 2-hydroxyglutarate (87). The accumulation of these oncometabolites promote cancer in various ways, including stabilization of HIF1 and DNA hypermethylation via inhibition of α-ketoglutarate-dependent dioxygenases [as reviewed in (48, 84)].

## Metabolism and drug resistance

It is becoming increasingly clear that changes in metabolism influence drug response to established first-line chemotherapy in several cancers, identifying metabolic rewiring as a novel, and important mechanism of adaptive resistance. Table 1 gives a comprehensive overview of studies linking metabolism to drug resistance in cancer.

**Table 1:** Overview of metabolic alterations associated with drug resistance in cancer.

**Cancer type**	**Pathways associated with resistance**	**Therapy**	**Target in resistance (proposed therapy)**	**References**
**MULTIPLE MYELOMA**				
	Glycolysis	Bortezomib	LDHA, PDK1	(88–91)
	Mitochondrial energy metabolism	Bortezomib, Carfilzomib	SOD2 (2ME),	(92–95)
	Redox metabolism	Bortezomib	–	(91, 93, 96)
	Glutaminolysis	Bortezomib, Carfilzomib	GLS (CB-839)	(92)
	Mevalonate pathway	Bortezomib	HMG-CoA (simvastatin)	(97)
	Serine synthesis	Bortezomib	PHGDH	(91)
	Pentose phosphate pathway	Bortezomib	–	(91)
**LUNG CANCER**				
	Mitochondrial energy metabolism	Cisplatin	–	(98)
	Glutaminolysis	Cisplatin	xCT antiporter (riluzole)	(98, 99)
	Redox metabolism	Cisplatin	ASS, TRX1 (Elesclomol), GCLC	(100–103)
	Glycolysis	Cisplatin, Paclitaxel, Carboplatin	HK2 (2-DG), mTOR, PDK2 (DCA), PKM2 (metformin)	(99, 104–106)
**OVARIAN CANCER**				
	Glycolysis	Cisplatin	2-DG	(107, 108)
	Pentose phosphate pathway	Cisplatin	G6PD (6-AN)	(107, 109)
	Redox metabolism	Cisplatin	GCLC (BSO), TRX (auranofin)	(107, 108)
	Fatty acid synthese	Cisplatin	FASN (orlistat)	(110)
	Glutaminolysis	Cisplatin, Paclitaxel	GLS (BPTES)	(110, 111)
**BREAST CANCER**				
	Fatty acid synthese	Adriamycin	FASN (Orlistat)	(112)
	Redox metabolism	Tamoxifen	GCLC (BSO)	(75, 113)
	Glycolysis	Lapatinib, Paclitaxel, Trastuzumab, Tamoxifen	HK (2-DG) LDHA (oxamate)	(52, 114–117)
	Mitochondrial energy metabolism	Lapatinib, Tamoxifen	ERRα, NQO1	(113, 118, 119)
**MELANOMA**				
	Mitochondrial energy metabolism	BRAF inhibitor	ETC (esclomol)	(120–123)
	Glutaminolysis	BRAF inhibitor	GLS (BPTES)	(120)
	Arginine metabolism	BRAF inhibitor	ASS1 (arginine starvation)	(124)
	Glycolysis	BRAF inhibitor (Vemurafenib)	PDK1 (DCA)	(121, 125)
	Redox Metabolism	BRAF inhibitor (Vemurafenib)	NAMPT	(126)
**PANCREATIC CANCER**				
	Glycolysis	Gemcitabine	FBP1	(127, 128)
	Fatty acid synthesis	Gemcitabine	FASN (orlistat)	(129, 130)
	Glutaminolysis	Gemcitabine		(131)
	Redox metabolism	Gemcitabine	xCT antiporter	(132)
	Pyrimidine synthesis	Gemcitabine	DODH (leflunomide)	(128)
**LEUKEMIA**				
	Glycolysis	Daunorubicin, Imatinib	PFK2	(133, 134)
	Mitochondrial energy metabolism	Imatinib, Cytarabine	–	(135, 136)
**HEPATOCELLULAR CARCINOMA**				
	Glutaminolysis	Sorefanib	GLS1 (BPTES), PPARδ	(137)
**SQUAMOUS CELL CARCINOMA**				
	Glycolysis	Cisplatin, radiation therapy	PKM2	(138, 139)
	Redox metabolism	Cisplatin, radiation therapy		(138, 139)
	Nucleotide metabolism	Gemcitabine	(metformin)	(140)
	Fatty acid synthesis	Radiation therapy	FASN (orlistat)	(139)
**COLON CANCER**				
	Glycolysis	Multiple chemotherapeutic agents		(141)
**ANAPLASTIC THYROID CANCER**				
	Pentose phosphate pathway	Doxorubicin	6PGD	(142)

In this section, we provide a more in-depth analysis of the four cancer-drug combinations on which most research has been done: Proteasome inhibitors for multiple myeloma, EGFR inhibitors for breast cancer, cisplatin for lung and ovarian cancer, and BRAF inhibitors for melanoma.

### Metabolism is linked to anticancer drug resistance to proteasome inhibitors

Proteasome inhibitors are a cornerstone in the treatment of multiple myeloma (143, 144), with bortezomib being the first clinically available proteasome inhibitor. Proteasome inhibition results in a disbalance between the production and degradation of proteins and eventually causes apoptosis in malignant cells via multiple pathways, including overproduction of ROS (145–147). Although bortezomib therapy prolongs survival, some patients show intrinsic resistance to therapy, while others develop resistance during treatment (21, 147, 148). Bortezomib resistance is associated with mutations in the proteasomal bortezomib-binding pocket and upregulation of the proteasomal machinery, both of which lower the efficacy of the drug (149–154). However, intracellular concentrations of bortezomib seem to correlate with proteasome inhibition, but not cytotoxicity (155). This suggests that adaptive resistance mechanisms are involved, which allow cells to proliferate even when proteasome function is impaired. Indeed, recent studies suggest that compensating mechanisms downstream of the proteasome are altered in bortezomib resistance, such as the unfolded protein response and vesicular exocytosis of ubiquitinated proteins (145, 156–159).

**Figure 2:**
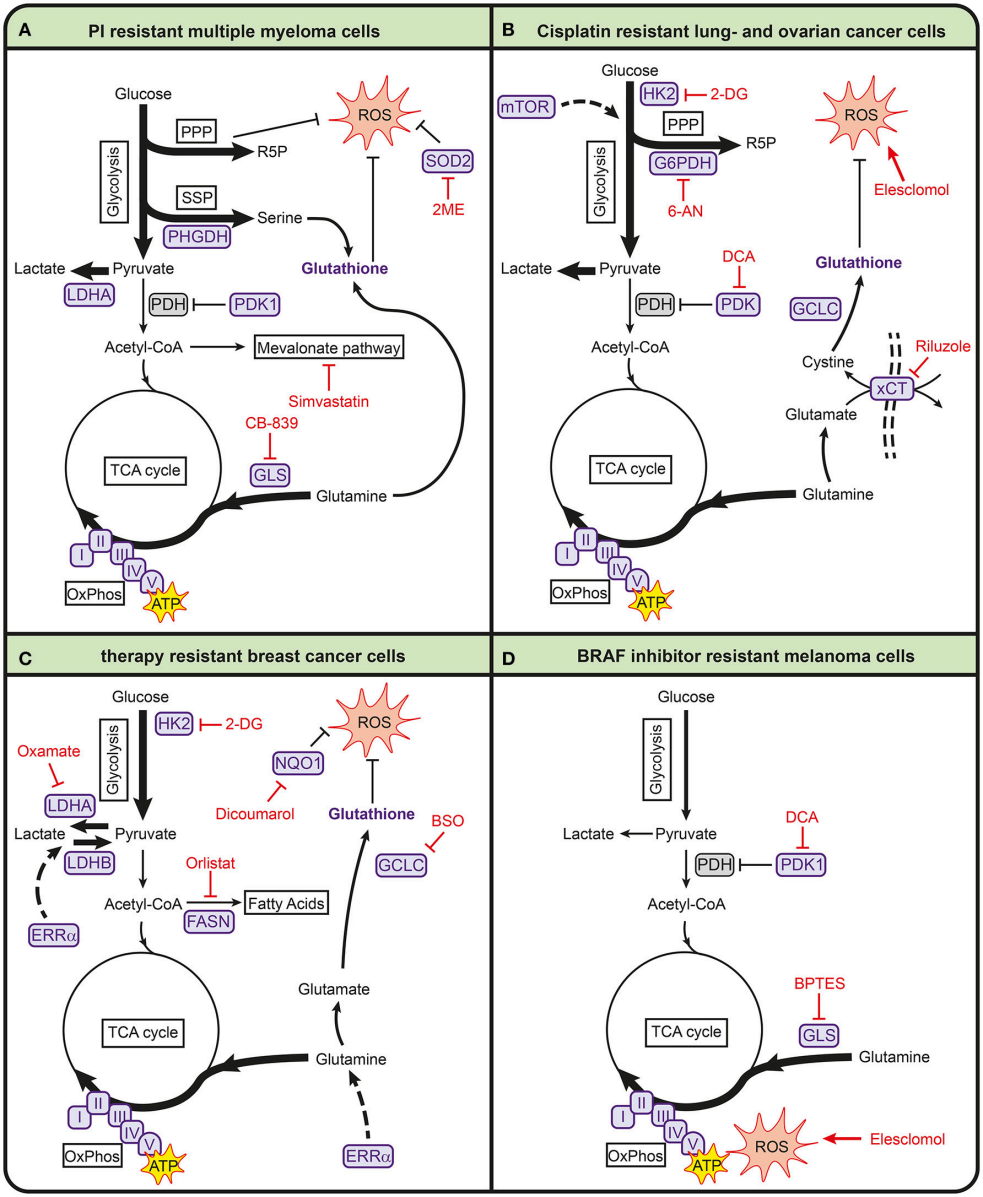
Metabolic pathways involved in anticancer drug resistance in multiple myeloma **(A)**, lung- and ovarian cancer **(B)**, breast cancer **(C)**, and melanoma **(D)**. Metabolic enzymes that are associated with drug resistance are shown in purple. Metabolic inhibitors that can be used to target drug-resistant cancers are depicted in red. 2-DG, 2-deoxyglucose; 2ME, 2-methoxyestradiol; 6-AN, 6-aminonicotinamide; ATP, adenosine triphosphate; BPTES, bis-2-(5-phenylacetamido-1,3,4-thiadiazol-2-yl)ethyl sulfide; BSO, buthionine sulphoximine; FASN, fatty acid synthase; G6PDH, glucose-6-phosphate dehydrogenase; GCLC, glutamate-cysteine ligase; GLS, glutaminase; HK2, hexokinase 2; I, complex I; II, complex II; III, complex III; IV, complex IV; LDHA, lactate dehydrogenase A; LDHB, lactate dehydrogenase B; mTOR, mammalian target of rapamycin; NQO1, NAD(P)H quinone dehydrogenase; PDH, pyruvate dehydrogenase complex; PDK1, pyruvate dehydrogenase kinase 1; PHGDH, 3-phosphoglycerate dehydrogenase; PPP, pentose phosphate pathway; R5P, ribose 5-phosphate; ROS, reactive oxygen species; SOD2, superoxide dismutase 2; SSP, serine synthesis pathway; TCA, tricarboxylic acid cycle; V, complex V; xCT, glutamate/cysteine xCT antiporter.

Several recent studies describe that metabolic processes are also involved in mediating sensitivity toward bortezomib (see Table 1, Figure 2A). In particular, pathways involved in energy metabolism and the anti-oxidant response are associated with bortezomib resistance (88–90, 92–95). For example, bortezomib-resistant cells have higher mitochondrial function and expression of mitochondrial genes (94). Proteomic screening of bortezomib-resistant and -sensitive cell lines also showed that resistant cells have increased levels of proteins involved in mitochondrial function and the generation of reducing equivalents (93). These increased levels of mitochondrial proteins are accompanied with higher activity of OXPHOS in proteasome inhibitor resistant cell lines (92). In addition, higher expression levels of genes related to OXPHOS were found in patients that responded poorly to bortezomib (92). Together, these studies suggest that bortezomib-resistant cells are more dependent on OXPHOS than –sensitive cells, making it a promising target for bortezomib-resistance.

In addition, bortezomib-resistant cells have higher expression of superoxide dismutase 2 (SOD2) which is important for mitochondrial ROS clearing (94). The combination of SOD2 inhibition and bortezomib induces cell death in bortezomib-resistant multiple myeloma cells via mitochondrial ROS overproduction (95). Because oxidative stress plays a role in the mechanism of action of bortezomib, it is likely that resistance is accompanied with increased antioxidant capacity. In line with this, high intracellular glutathione levels protect cells from bortezomib-induced apoptosis (96). Other antioxidant-related pathways are also upregulated in bortezomib-resistant cells, such as the PPP and SSP (91). High levels of PHGDH were found in bortezomib-resistant cells and starving multiple myeloma cell lines for serine during bortezomib treatment enhanced bortezomib toxicity (91). This demonstrates the importance of serine metabolism in bortezomib resistance.

Finally, several studies show that bortezomib resistance is accompanied with higher glycolytic activity (88, 89, 91, 93). Soriano et al. showed that proteasome inhibitor-resistant cells display higher levels of glycolytic enzymes and higher glycolytic rates than parental cell lines (93). Higher glycolytic activity has also been found to lower bortezomib sensitivity under hypoxic conditions, while inhibition of LDHA enhances sensitivity of bortezomib under these conditions (89). In addition, LDHA expression correlated to poor prognosis in multiple myeloma patients (88). Zaal et al. showed that bortezomib-resistant cells have a higher uptake of extracellular glucose, which is used for biosynthetic pathways branching off from glycolysis to support a higher anti-oxidant capacity (91). This is in line with findings that showed that dichloroacetate (DCA), which inhibits PDK1 and thereby promotes pyruvate entry into the TCA cycle, increases sensitivity of multiple myeloma cells to bortezomib both *in vitro* and in myeloma-bearing mice (88, 90).

### Metabolic rewiring in lung and ovarian cancer in response to cisplatin treatment

Cisplatin is a widely used chemotherapeutic agent in several types of cancer, including lung cancer and ovarian cancer. Cisplatin interacts with reducing equivalents (e.g., GSH) and DNA, resulting in increased ROS and DNA damage, which eventually leads to apoptosis (160). Many mechanisms involved in cisplatin resistance have been described, including reduced cisplatin uptake, increased DNA repair mechanisms and anti-apoptotic pathways [reviewed in (20, 160)]. Several studies suggest that metabolic rewiring in cisplatin-resistant cells is involved in redox buffering in both lung cancer and ovarian cancer cells to counteract cisplatin therapy (Table 1, Figure 2B) (98, 100, 101, 103, 107). Cisplatin-resistant lung cancer cells have higher levels of ROS, in part due to low levels of intracellular thioredoxin (100), but display higher levels of GSH and GCLC (20, 103), likely to counteract the high ROS levels induced by cisplatin (161). Catanzaro et al. showed that cisplatin-resistant ovarian cancer cells have higher levels of GSH and G6PD and that PPP inhibition with 6-aminonicotinamide (6-AN) increases cisplatin cytotoxicity in these resistant cells (107, 109). In line with this, several studies show that cisplatin-resistant cells are vulnerable for ROS inducing agents. Cisplatin-resistant cells lung cancer cells have been reported to be more sensitive to elesclomol, an agent that is known to increase ROS (98). In addition, the xCT-cysteine/glutamate pump that provides cells with cystine for GSH synthesis, is upregulated in these cisplatin-resistant cells, and they are more sensitive to the xCT-cysteine/glutamate pump inhibitor riluzole as compared to their parental counterpart (98). Also, inhibition of GSH biosynthesis with buthionine sulfoximine (BSO) enhances the effect of cisplatin in breast cancer cells (75).

Cisplatin-resistant cells have an altered energy metabolism compared to sensitive cells, but the findings on glycolysis and oxidative phosphorylation in lung and ovarian cancers are opposing. Cisplatin-resistant ovarian and cervical cancer cells were found to have higher rates of glycolysis and reduced mitochondrial activity compared to their cisplatin-sensitive counterparts. This leads to a higher sensitivity of resistant ovarian cancer cells to glucose starvation or to treatment with 2 deoxyglucose (2-DG), a competitive inhibitor of HK (107, 108). Cisplatin-resistant lung cancer cells, on the other hand, have lower rates of glycolysis and instead rely on oxidative phosphorylation (98, 99). These lung cancer cells have lower levels of HK1 and HK2 (99), in accordance with the observation that cisplatin treatment itself lowers HK expression (162). Cisplatin-resistant lung cancer cells also display lower glucose uptake and lower levels of LDHA and lactate production as compared to sensitive parental cell lines (98), all indicative of a lower glycolytic activity. In line with lower glycolysis rates, cisplatin-resistant lung cancer cells are not sensitive to glucose starvation under normal growth conditions. However, under hypoxic conditions, these cells are more vulnerable for 2-DG treatment as compared to the parental cells. As cells depend on glycolysis for their energy production in the absence of oxygen, the lower levels of HK in cisplatin-resistant cells likely makes them more vulnerable for 2-DG under these conditions (99). The lower glycolytic activity in cisplatin-resistant lung cancer cells is accompanied by higher rates of oxidative phosphorylation and mitochondrial activity (98–100), as well as a higher dependence on glutamine (98). Also β-oxidation of fatty acids has been described to fuel the TCA-cycle in cisplatin-resistant lung cancer cells (99, 100). In line with these findings, inhibition of glutaminase sensitized cisplatin-resistant ovarian cancers to chemotherapy (110, 110) and also the inhibition of FASN with orlistat enhanced the efficacy of cisplatin in ovarian cancers (111).

Interestingly, the metabolic reprogramming in lung cancer cells seems to some extent specific for cisplatin. Lung cancer cells that are resistant to carboplatin, which has a similar mode of action, are more dependent on glycolysis (106). In addition, paclitaxel-resistant lung cancer cells show higher expression of PDK2 as compared to their parental cells (105). As a result, these resistant cells are more dependent on glycolysis than OXPHOS and could be sensitized to paclitaxel through PDK2 inhibition. These examples highlight the heterogeneity of metabolic alterations in response to drugs and indicate that these can not only be tumor specific, but also drug specific.

### Metabolic alterations involved in drug-resistant breast cancers

Many breast cancers overexpress the receptor tyrosine kinase ErbB2 and several drugs that target ErbB2, such as trastuzumab and lapatinib, are used in the treatment of breast cancer. Several mechanisms of resistance against these targeted therapies have been described, including the re-activation of downstream kinase pathways and oncogenic signaling (112, 163, 164). In addition, metabolism pays a role in mediating resistance against tyrosine kinase inhibitors.

Increased glycolysis is a common feature of drug-resistant breast cancer cells irrespective of the type of chemotherapeutical agent used (Table 1, Figure 2c), but this increased activity is regulated in different ways in different resistant breast tumors. For example, several studies have found that resistance to lapatinib is associated with increased glycolysis (114, 115). Lapatinib-resistant SKBR3 breast cancer cells showed increased expression of genes associated with glucose deprivation compared to sensitive cells, which correlated to poor outcome in patients. These genes included glucose transporters and glycolytic enzymes, as well as alternative pathways for energy production, such as β-oxidation (114). Higher glycolytic activity and an increased sensitivity toward inhibition of glycolysis were also found in lapatinib-resistant BT474 breast cancer cells using a multi-omics approach involving (phospho)proteomics and metabolomics (115). Interestingly, the higher glycolytic rates in these BT474 cells were not resulting from higher expression levels of glycolytic enzymes, but merely from changes in the phosphorylation state of glycolytic enzymes, showing that post-translational modifications alone can regulate glycolytic activity. In trastuzumab-resistant ErbB2-positive breast cancer cells, increased glycolytic activity is mediated by heat shock factor 1 and LDHA and inhibition of glycolysis with 2-DG and the LDH inhibitor oxamate resensitizes resistant cells to trastuzumab (117). Finally, in paclitaxel-resistant breast cancer cells, synergistic effects on promoting apoptosis were observed when LDHA was genetically downregulated or when paclitaxel was combined with oxamate (116).

Interestingly, Park et al. showed that the nuclear receptor estrogen-related receptor alpha (ERRα) regulates a metabolic switch to allow breast cancer cells to use lactate as a substrate for mitochondrial respiration in the absence of glucose. The ability to bypass glycolysis makes these cells less vulnerable for PI3K/mTOR inhibitors in the presence of lactate and ERRα antagonists are able to restore drug efficacy (119), underscoring the importance of nutrient availability drug efficacy. The importance of ERRα in regulating metabolism is further emphasized by Deblois et al., who showed that lapatinib-resistant breast cancer cells restore ERRα levels through reactivation of mTOR signaling, resulting in increased glutamine metabolism, mitochondrial energy production and anti-oxidant capacity (118). Moreover, in a HER2-induced mammary tumor mouse model, targeting ERRα counteracts the metabolic alterations associated with lapatinib resistance and overcomes resistance to this drug (118). Targeting ERRα is therefore emerging as a strategy to increase the sensitivity of drug-resistant breast cancer cells in the context of metabolism.

Another metabolic aspect that is observed in drug-resistant breast cancer cells is increased levels of OXPHOS, coupled to higher levels of oxidative stress. For example, tamoxifen-resistant MCF-7 breast cancer cells display increased mitochondrial metabolism and ATP production (113). The biguanides metformin and phenformin, which inhibit ETC, selectively kill breast cancer stem cells that were resistant to standard chemotherapy (165), underscoring the importance of OXPHOS activity in drug response. This higher mitochondrial activity may also explain the observation that tamoxifen-resistant breast cancer cells display lower levels of GSH (113), as these cells probably experience higher levels of oxidative stress. In line with this, tamoxifen-resistant cells have higher expression of NADPH dehydrogenase 1 (NQO1) and GCLC, both involved in the defense against oxidative stress. Moreover, transduction of these genes into MCF-7 cells results in a tamoxifen-resistant phenotype and NQO1 mRNA levels associate with disease progression in patients that received endocrine therapy. As a result, NQO1 inhibition with dicoumarol restored tamoxifen sensitivity in tamoxifen-resistant breast cancer cells (113). Increased GSH synthesis was also observed in PI3K/Akt driven breast cancer and required for resistance to oxidative stress, Inhibition of GSH biosynthesis with BSO synergized with cisplatin to induce regression of in PI3K/Akt driven breast cancer (75). Together, these results suggest that an increased anti-oxidant defense mechanism drives resistance in breast cancer to various types of chemotherapy.

### Metabolic contributions to BRAF inhibitor resistance in melanoma

Most cutaneous melanomas harbor activating mutations in the protein kinase BRAF, which makes inhibitors that target mutant BRAF promising agents to treat melanoma patients. In terms of metabolism, melanomas that express mutant BRAF and that developed resistance against BRAF inhibitors display increased activity of mitochondrial oxidative metabolism, increased dependency on mitochondria for survival and higher levels of ROS (Table 1, Figure 2D) (120–123, 126). For example, treatment of mutant BRAF melanoma cells with the BRAF inhibitor vemurafenib results in increased mitochondrial respiration. Inhibition of mitochondrial respiration enhances vemurafenib-induced cell death, suggesting that increased mitochondrial activity serves as a defense mechanism against the drug. At the same time, increased levels of ROS that accompany the increased respiration renders these cells more vulnerable for further oxidative stress induced by exogenous agents such as elesclomol (122). Baenke et al. showed that the increased dependency on mitochondrial respiration is associated with a metabolic switch that makes cells more dependent on glutamine rather than glucose. Hence, resistant BRAF mutant melanoma cells are more sensitive to the GLS inhibitor BPTES, which reduces ATP levels in resistant cells but not in parental cells. Moreover, BPTES enhances the anti-tumor activity of BRAF inhibitors, underscoring the importance of glutamine in mediating BRAF inhibitor resistance (120). A second metabolic switch in BRAF inhibitor-resistant melanoma cells was found on the level of PDK (121, 125). PDK inhibition reduced viability of BRAF inhibitor resistant cells, likely by increasing pyruvate influx into the TCA cycle and thereby mitochondrial ROS (121). The susceptibility to higher levels of oxidative stress was also observed in other tumor types that harbored a mutation in BRAF, as mutant BRAF colorectal cancer cells are prone to cell death after exposure to the oxidized form of vitamin C, which causes oxidative stress via GSH depletion (166).

## Targeting drug resistance through the manipulation of metabolism

From the studies discussed above, it becomes apparent that anticancer drug resistance to first-line chemotherapy is often linked to metabolic alterations and consequently, that these may be targeted to overcome drug resistance or to enhance the efficacy of current chemotherapy. Among the different drug-resistant cancers described, pathways involved in redox and energy metabolism are frequently altered (Table 1, Figure 2), making them promising pathways to target drug-resistant cancers.

Resistance to several anticancer agents, including proteasome inhibitors, cisplatin, EGFR inhibitors and BRAF inhibitors, is accompanied with increased activity of pathways involved in redox balance, suggesting that interfering with redox metabolism can improve response to a wide range of drugs and aid in overcoming multidrug resistance. A majority of anticancer agents induce apoptosis by increasing oxidative stress (96, 101, 132, 137, 167–169) and it is likely that drug-resistant cells in general increase their anti-oxidant capacity to counteract the effect of drug treatment, albeit via different pathways. But although an increased anti-oxidant capacity seems to be a common characteristic of drug-resistant cells, metabolic profiles are altered in drug-specific manners. For example, bortezomib- and sorafenib-resistant cells as well as cisplatin-resistant ovarian cancers rely more on NADPH production via the PPP (91, 107, 137), while tamoxifen-resistant cells and cisplatin-resistant lung cancers have higher activity of GSH synthesis (75, 103, 113). The fact that several different pathways are involved suggests not only that a tailored approach may be needed to overcome resistance to specific drugs, but also that these redundant pathways may protect cancer cells to a large extent from inhibition of one specific pathway.

In addition, many studies show an association between drug resistant cells and the Warburg effect, suggesting that a high glycolytic rate helps cancer cells to survive anticancer treatment, such as bortezomib, cisplatin and lapatinib (89, 116, 121, 125, 170). As a consequence, glycolytic inhibition with 2-deoxyglucose could be a novel strategy to overcome drug resistance. It has been postulated that higher glycolytic rates may lower drug efficacy through the increased secretion of lactate and acidification of the extracellular space, as some drugs are not stable under acidic conditions (171, 172). High glycolytic rates in drug-resistant cells are often accompanied with higher expression of glycolytic regulators such as PDK1 and LDHA, making these enzymes interesting targets for drug-resistant cancers. In contrast, bortezomib- and BRAF inhibitor-resistant tumors rely more on mitochondrial activity fuelled by glutamine rather than glucose (92, 98, 120). Interfering with glutamine metabolism, via either inhibition of glutaminolysis or glutamine uptake, could be a strategy for drug-resistant tumors that rely on glutamine (173, 174). In addition, as glutamine is mainly used for mitochondrial energy production, inhibition of the ETC with biguanides, such as metformin and phenformin, holds great promise in cancer therapy and drug resistance (173, 175).

Finally, most studies on metabolism-mediated drug resistance have so far focused on glycolysis and the TCA cycle and on the role glucose and glutamine. But fatty acids and branched chain amino acids can also provide energy and are also linked to tumorigenesis (77, 176). Interesting opportunities to target drug resistance may therefore also be found beyond glycolysis and the TCA cycle. FASN correlates with poor prognosis in various types of cancer and also interferes with drug efficacy (77). FASN overexpression causes resistance to the anticancer drugs adriamycin and mitoxantrone in breast cancer cells (112), gemcitabine-resistant pancreatic cells (129), cisplatin-resistant ovarian cancer cells (110), and radiotherapy resistant head and neck squamous cell carcinomas (139). Orlistat, a FASN inhibitor, increases the sensitivity to all drugs, suggesting that FASN can be a new target in drug resistant cancers. Amino acid metabolism may also yield promising targets to treat drug-resistant tumors. Cancer cells can be dependent on specific amino acids, such as serine (58, 177), proline (74, 178), aspartate (65, 73), and arginine (179). Although the role of amino acid metabolism in drug resistance is largely unexplored, studies suggest that amino acid availability could be important in drug response and the development of drug resistance. For example, BRAF inhibitor-resistant melanoma cells are more sensitive to arginine deprivation as compared to parental cells (124). Finally, serine synthesis is associated with bortezomib resistance in multiple myeloma and serine starvation enhanced the cytotoxic effect of bortezomib (91). These studies not only underscore the complexity of cancer metabolism, but also suggest that the exploration of amino acid metabolism may be a promising avenue to identify novel targets to overcome drug resistance.

## Conclusions

It is clear that understanding cancer metabolism can improve cancer therapy, as exemplified by the widespread use of 18-fluorodeoxyglucose, a glucose analog that exploits the Warburg effect in PET imaging for cancer diagnosis, treatment, and prognosis (180). The potential of metabolic inhibitors in cancer is also illustrated by the use of antimetabolites, such as 5-fluorouracil and methotrexate, which have been used for decades to treat cancers (181), even though their anticancer effects were only coupled to metabolic interference much later (14). Another example of a successful metabolic drug is L-asparaginase, which is used in the treatment of acute lymphoblastic leukemia (182). The recent surge in knowledge in the field of cancer metabolism has sparked increased interest to exploit the altered metabolism of cancer cells to find novel targets for therapy. As a result, compounds have been developed that specifically target the unique metabolism of cancers. Several of these compounds targeting for example glycolysis, TCA cycle and OXPHOS are now being tested in clinical trials (13, 14, 160, 183).

In this review, we discussed specific metabolic programs and adaptations that exist in drug-resistant tumors, how these adaptations depend both on the drug and the origin of the tumor and how they contribute to drug resistance. From these studies, it becomes apparent that for many first-line chemotherapeutic agents, combinational treatments with metabolic drugs hold great promise to increase drug efficacy. Moreover, a better understanding of the altered metabolism in different drug resistant cancers is essential to further improve cancer therapy. Such understanding will provide insights into the molecular mechanisms of resistance to identify novel metabolic targets that can be used for (combinational) therapy. Finally, this knowledge may also lead to prognostic biomarkers for drug response, which could advance current therapy by predicting drug response based on the metabolic state of a tumor and thereby contribute toward more efficacious personalized medicine.

## Author contributions

CB and EZ wrote the manuscript. Both authors read and approved the final version of the manuscript.

### Conflict of interest statement

The authors declare that the research was conducted in the absence of any commercial or financial relationships that could be construed as a potential conflict of interest.

## Abbreviations

2-DG: 2-deoxyglucose
6-AN: 6-aminonicotinamide
ATP: adenosine triphosphate
BSO: buthionine sulfoximine
CPT1: carnitine palmitoyl transferase 1
DCA: dichloroacetate
ETC: electron transport chain
FASN: fatty acid synthase
FH: fumarate hydratase
G6PD: glucose-6-phosphate dehydrogenase
GCLC: glutathione cysteine ligase
GLS: glutaminase
GLUT: glucose transporter
GSH: glutathione
HIF1: hypoxia inducible factor 1
HK2: hexokinase 2
IDH1: isocitrate dehydrogenase 1
IDH2: isocitrate dehydrogenase 2
LDHA: lactate dehydrogenase A
MCT4: monocarboxylate transorter 4
ME: malic enzyme
NQO1: NADPH dehydrogenase
OAA: oxaloacetate
OXPHOS: oxidative phosphorylation
PDK1: pyruvate dehydrogenase kinase 1
PFK2: phosphofructokinase 2
PGAM1: phosphoglycerate mutase 1
PHGDH: 3-phosphoglycerate dehydrogenase
PKM2: pyruvate kinase isoform M2
PPP: pentose phosphate pathway
ROS: reactive oxygen species
SDH: succinate dehydrogenase
SOD2: superoxide dismutase 2
SSP: serine synthesis pathway
TCA: tricarboxylic acid.
